# Trigeminal ganglion itself can be a viable target to manage trigeminal neuralgia

**DOI:** 10.1186/s10194-022-01512-x

**Published:** 2022-11-24

**Authors:** Elina KC, Jaisan Islam, Young Seok Park

**Affiliations:** 1 Program in Neuroscience, Department of Medicine, College of Medicine, Chungbuk National University, Cheongju, Korea; 2 Department of Neurosurgery, Chungbuk National University Hospital, Cheongju, Korea

**Keywords:** Headache, Neuromodulation, Neuropathic pain, Trigeminal ganglion

## Abstract

Excruciating trigeminal neuralgia (TN) management is very difficult and severely affects the patient’s quality of life. Earlier studies have shown that the trigeminal ganglion (TG) comprises several receptors and signal molecules that are involved in the process of peripheral sensitization, which influences the development and persistence of neuropathic pain. Targeting TG can modulate this sensitization pathway and mediate the pain-relieving effect. So far,there are few studies in which modulation approaches to TG itself have been suggested so far. “Trigeminal ganglion modulation” and “trigeminal neuralgia” were used as search phrases in the Scopus Index and PubMed databases to discover articles that were pertinent to the topic. In this review, we address the role of the trigeminal ganglion in TN and underlying molecules and neuropeptides implicated in trigeminal pain pathways in processing pathological orofacial pain. We also reviewed different modulation approaches in TG for TN management. Furthermore, we discuss the prospect of targeting trigeminal ganglion to manage such intractable pain.

## Background

Trigeminal neuralgia (TN), commonly referred to as tic douloureux, is a severe facial condition marked by intense, quickly passing stabbing pain attacks that are elicited by stimuli [1]. The trigger factors of TN include routine activities like chewing, talking, and brushing. Small sensory-sensitive zones are where these triggers are situated. The sites of a sensory trigger and the sites of pain, however, are not always the same [2]. The major pathophysiological mechanisms of TN include compression of the trigeminal nerve root, primary demyelinating disease, and central pain-related circuit sensitization and dysfunction. The hyperactivity or aberrant discharge from the injured nerve roots, ganglion, and trigeminal nucleus in the brainstem is speculated to be accountable for the pain. Medication, surgery, and complementary treatment approaches are offered. Medical pharmacotherapy is the preferred initial treatment for TN. Carbamazepine and oxcarbazepine (sodium channel blockers) are the first-line medications. Other cornerstones of treatment are drugs like tricyclic antidepressants and anticonvulsants [3]. Despite being the most prevalent neuralgia, it is a rare condition. TN still presents difficulties for healthcare professionals as efficient, safe, and well-established therapies cannot be undertaken till a precise diagnosis is made. Dental pain, migraine, cluster headaches, and temporomandibular joint disorders are all common misdiagnoses [4, 5]. Nerve injections with local anesthetics or steroids may provide temporary pain relief for certain people, but most of the time, the relief is just momentary. In the past, neurosurgical destructive and ablative procedures like neurotomies, neurolysis, and surgical decompression have been frequently utilized. These methods could, however, lead to issues like the emergence of delayed deafferentation pain in the vicinity of the injured nerve. The related morbidity and challenges with pain recurrence restrict these irreversible procedures' long-term effectiveness [6]. Technological advances in neuromodulation have offered such refractory conditions a phenomenal perspective [7].

The trigeminal ganglion (TG) is a crucial element in the nociception of craniofacial pain and plays a role in the peripheral modulation of pain pathways in the TN [8]. Damaged TG neurons brought on by unintentional and spontaneous nerve lesions are known to induce severe pain and sensory abnormalities. TG had been thought to be a node of transition for sensory data commuting from the periphery to the center. The fact that it can function as an integrating structure in the peripheral nervous system to control intracellular modulation mechanisms, as well as intercellular and autocrine signaling, is now well documented [9]. Targeting this essential component of the trigeminal pain pathway has been increasingly popular in recent years. A thorough review is necessary to offer information on its potential.

In this review, PubMed and Scopus Index were used as major search engines to find relevant articles on trigeminal ganglion modulation approaches in trigeminal neuralgia. The preliminary search retrieved 34 non-duplicated entries from the database upon implementing the key terms without accounting for the publication date. Only 25 of them were significantly relevant to the subject and featured in this review. Additionally, we referenced literature from the Scopus index that was compiled for clarity concerning the TG and trigeminal pain circuitry.Here, we summarize the trigeminal ganglion-focused neuromodulation techniques employed to address trigeminal neuralgia. These studies demonstrate the significance of the trigeminal ganglion in TN, alterations to the TG following TN, multiple approaches for targeting the TG, and its prospects. As a potential therapeutic strategy for trigeminal neuralgia, we propose the targeted modulation of the trigeminal ganglion itself.

## Characteristics of Trigeminal ganglion

The TG is a sensory ganglion that resides in the dura mater's Meckel cave. The ocular (V1), maxillary (V2), and mandibular (V3) nerves, which in rodents form a single thick bundle at their origin, are generated by the TG. Most of the peripheral axons of pseudo-unipolar primary afferent neurons are found in those three large cranial nerves [10]. The source of the central processes of the trigeminal afferents is the TG, which produces the trigeminal nerve and enters the brainstem at the pontine level. The TG is made up of glial cells and neurons[11]. The distinctive pseudounipolar architecture of TG neurons interjected links between the two terminals and the discharge of transmitters at both central and peripheral sites [12]. TG neurons can be categorized into two sizes: large and small. Based on established studies, the modulation of pain in TN is mediated by small-sized TG neurons that are mostly coupled to unmyelinated nerve fibers [13]. The glial cells that surround the pseudo-unipolar neurons have a substantial impact on the activity and function of the neurons by altering the ionic concentrations [14].

## Neuropeptides and their receptors signaling in TG

TG expresses a wide range of neuropeptides. Due to their role in the trigeminal pain pathway, these neuropeptides are of great interest. Distinct neuropeptides secreted by TG are calcitonin gene-related peptide (CGRP), substance P (SP), and vasoactive intestinal peptide (VIP) [15]. Numerous additional neuropeptides have been outlined by immunostaining studies in TG, including neurokinin A, pituitary adenylate-cyclase-activating polypeptide receptor type 1 (PACAP), cholecystokinin, galanin, somatostatin, and opioid peptides. They contribute to signal transmission by serving as neuromodulators [16].

About 40–50% of the trigeminal system's neurons are CGRP-immunoreactive, making it the most dominant signaling neuropeptide in the region [17]. There are two isoforms of CGRP: α- and β-CGRP. Among them, α-CGRP predominates in sensory nerves with cell bodies in the dorsal root ganglion (DRG) and TG [18]. CGRP receptor expression in the TG is implicated in signaling pathways that could be crucial for sensitizing processes in the induction of headaches and facial pain [19]. TG neurons express transient receptor potential (TRP) family receptors in substantial concentrations. In most TG neurons, immunoreactivity for the TRP vanilloid type 1 receptor channel (TRPV1) has been found [20]. 10–30% of TG cells are SP-containing cells, which are dispersed throughout the region. TG neurons also have a high affinity for the SP receptor, neurokinin 1-receptor (NK_1_-R) [21]. VIP-positive cells make up 10–12% of TG neurons and are linked to TG cells that contain SP. A subpopulation of small-to medium-sized TG neurons contains PACAP, and members of the family of seven-transmembrane G protein-coupled receptors mediate its effects [22]. Only a small fraction of TG neurons are reactive to neuropeptide Y (NPY) as it is primarily produced in sympathetic neurons [23].

Orexins and oxytocin are additional neuropeptides that may participate as signals in the TG but are most likely derived from different sources. In rat TG neurons, orexin receptor (OX1R and OX2R) mRNA has been identified, of which the OX _2_ R mRNA level was more abundantly expressed than the OX _1_ R [24]. TG neurons have also been found to exhibit oxytocin receptor immunoreactivity [25].

## Alterations in TG after trigeminal nerve injury

As per earlier research, the fundamental abnormality is centered in the TG rather than the skin or the central nervous system, and the sensation is substantially typical in between episodes of ectopic paroxysmal ganglion discharge [26]. Trigeminal nerve damage significantly alters the plasticity of TGs' biomarkers. Major alterations in injured TGs point to the possibility that NPY and Iba1 may be key players in the pathophysiology of orofacial neuropathic pain because of peripheral nerve injury [27]. The TG's parts V1, V2, and V3 are associated with each other, and stimulation of the V3 neurons may raise the amounts of active signaling proteins in the satellite glial cells (SGC) and neuronal cells in other ganglion regions, which aids in signal transmission and chronic pain. SGC undergoes modifications like an increase in gap junctions and the building of bridges that bind the perineuronal sheaths [28].

Following a trigeminal nerve injury, neuropeptides (such as SP and CGRP), several neurotrophic factors, glutamate, and ATP impact neuronal activity in both intact and injured TG neurons. Both the ganglion and the nerve are susceptible to these variations. The amplification of the perceptive neuronal excitability in the TG is facilitated by the active cell-to-cell interface among macrophages and TG neurons via various chemical messengers. As per reports, peripheral nerve damage triggers action potentials defined as injury discharges in the TG neurons, which are conveyed to the trigeminal spinal nucleus (Vc) [29].

## Trigeminal ganglion stimulation approaches

**Fig. 1:**
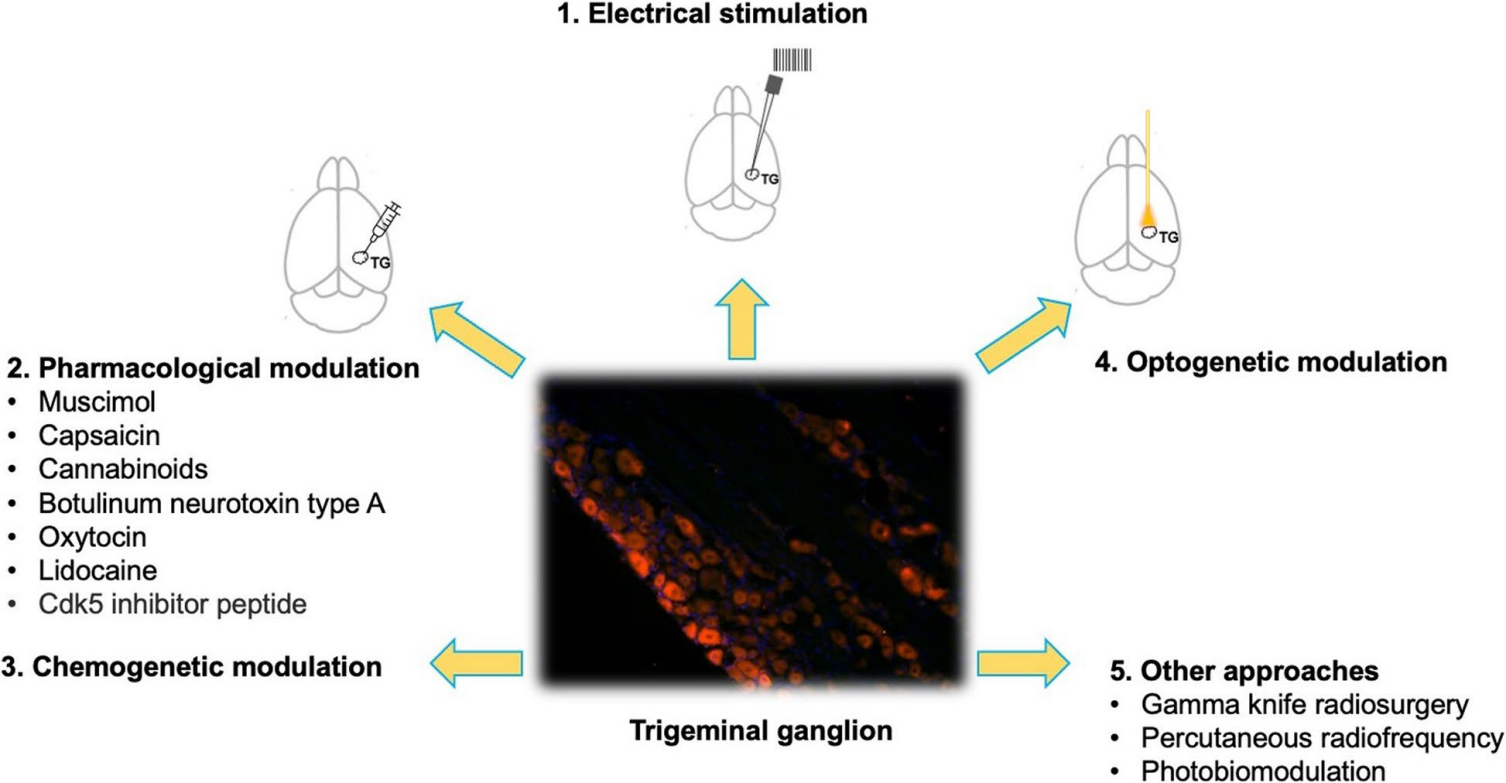
Different trigeminal ganglion stimulation approaches for managing TN pain

Addressing the trigeminal nerve central terminal for trigeminal pain relief has been undertaken in a multitude of ways. It's an intriguing strategy to target the trigeminal ganglion itself. Below, we've explored various trigeminal ganglion stimulation approaches made to date for managing TN pain (Fig. 1 and Table 1).

**Table 1:** Trigeminal ganglion stimulation approaches in orofacial neuropathic pain

Study	Modulation approach	Subject	Condition	References
Evaluation of c-Fos immunoreactivity in the rat brainstem nuclei relevant in migraine pathogenesis after electrical stimulation of the trigeminal ganglion	Electrical stimulation	Rat	Migraine	[30]
Trigeminal neuropathy: Two case reports of Gasserian ganglion stimulation	Electrical stimulation	Human	Refractory trigeminal neuropathy	[31]
Case Report: Novel Anchoring Technique and Surgical Nuances for Trigeminal Ganglion Stimulation in the Treatment of Post-Herpetic Trigeminal Neuropathic Facial Pain	Electrical stimulation	Human	Post-herpetic trigeminal neuropathic pain	[32]
Chronic electrostimulation of the trigeminal ganglion in trigeminal neuropathy: current state and future prospects	Electro stimulation	Human	Trigeminopathic pain	[33]
Activation of oxytocin receptor in the trigeminal ganglion attenuates orofacial ectopic pain attributed to inferior alveolar nerve injury	Pharmacological activation	Rat	Orofacial ectopic pain	[34]
Oxytocin alleviates orofacial mechanical hypersensitivity associated with infraorbital nerve injury through vasopressin-1A receptors of the rat trigeminal ganglia	Pharmacological modulation	Rat	Trigeminal nerve injury	[35]
Intra-arterial Modulation of the Trigeminal Nerve Ganglion in Patients with Refractory Trigeminal Neuralgia	Pharmacological modulation	Human	Refractory trigeminal neuralgia	[36]
Chemogenetic inhibition of trigeminal ganglion neurons attenuates behavioural and neural pain responses in a model of trigeminal neuropathic pain	Chemogenetic inhibition	Rat	Trigeminal neuropathic pain	[37]
Pain relief in a trigeminal neuralgia model via optogenetic inhibition on trigeminal ganglion itself with flexible optic fiber cannula	Optogenetic inhibition	Rat	Trigeminal Neuralgia	[38]

### Electrical stimulation of TG

Few clinical studies have reported the analgesic effect in TN patients following electrostimulation of TG. The potential of TG stimulation to access multiple dermatomes with a single electrode renders it feasibly interesting. In a clinical trial study, it has been advised that patients who experience chronic trigeminopathic pain should always consider persistent therapeutic electrostimulation of the TG [33]. In certain studies, electrical stimulation of the trigeminal ganglion influences both the peripheral and the central terminals of the primary trigeminal sensory neuron. In response to the nociceptive input, it is postulated that the stimulation triggers the ascending nociceptive routes to the thalamus and the cortex, which then activate the descending modulatory system [30]. The TG, also known as the Gasserian ganglion, is a conjunction of all three trigeminal branches that permits one to designate precise facial areas based on the available somatotopic arrangement of nerve fibers while achieving a therapeutic effect on the full half of the face with minor surgical intervention. In refractory trigeminal neuropathy, stimulation of the Gasserian ganglion is an effective, minimally invasive, and non-destructive treatment that ought to be considered earlier in the treatment algorithm [31]. Despite a few risks, such as electrode dislocation (10–30%) and other mechanical defects (24%), TG electric stimulation has shown to be effective in treating patients with chronic trigeminal neuropathic pain and persistent idiopathic facial pain who have tried and failed or were not deemed candidates for standard surgical interventions [39].

### Pharmacological modulation

Pharmacological approaches have also been made in TG to intervene in TN management. According to in vivo research by Minghan et al.,a Cdk5 inhibitor peptide, TFP5, can directly affect peripheral nerve pain signaling at the TG.In addition to reducing the polymodal nociceptor response to capsaicin and brush, direct treatment of TG with TFP5 significantly reduced integrated calcium signaling for both stimuli. Inhibition of Cdk5 reduces pain at the peripheral level via reducing pain signaling in peripheral TG neurons. Cdk5 activity at TG’s main sensory neurons could be the basis of more efficient and secure therapies for facial pain [40]. A different study used the organ culture method to investigate if botulinum neurotoxin A can directly interact with sensory processes in the trigeminal ganglion [41].

Another study revealed that oxytocin, a neuropeptide hormone, is crucial in mitigating TG neuronal hyperexcitability following nerve injury. The action is most likely attributed to vasopressin-1A receptor-mediated regulation of voltage-gated potassium channels [35]. Likewise, another group documented that the intraganglionic injection of oxytocin attenuated orofacial ectopic pain produced by inferior alveolar nerve transection. Targeting oxytocin receptors (OXTR) in TG may be a viable therapeutic strategy for treating chronic orofacial neuropathic pain since activation of OXTR in TG can diminish aberrant pain by inhibiting the action of CGRP, interleukin-1β, and tumor necrosis factor-α [34].

A clinical report also indicated that in patients with refractory TN, intra-arterial anesthetic injection to the TG could be employed to regulate trigeminal nerve activity. In the region of the middle meningeal artery close to the arterial branch serving the trigeminal nerve ganglion, researchers delivered intra-arterial lidocaine of up to 50 mg persession. The authors stated that consistent intraprocedural electrophysiologic suppression and short-term clinical improvement in subjects with resistant TN support the possibility of modulating trigeminal nerve activity via the arterial route [36].

### Optogenetic modulation of TG

The technique of optogenetic modulation in TG is new and elusive in the literature. Not long ago, we published an article reporting that manipulating TG activity directly through an optogenetic approach might govern the varied attributes of TN pain sensations and elicit antinociception. We have demonstrated that thalamic relay nuclei and the trigeminal brainstem, which direct to the somatosensory cortex, are modified by halorhodopsin-mediated inhibition of TG. Additionally, we highlighted that the inhibition has a significant impact on bursting activity and that thehyperpolarization results in a general reduction in thalamic discharge [38].

### DREADD activation of TG

Notably, Korczeniewska and the team approached the TG modulation with systemic administration of the DREADD agonist clozapine N-oxide (CNO). They claimed that by intervening in TG cells that are hyperactive, CNO-mediated activation of hM4Di-DREADDs in the TG improves nerve injury-induced neuropathic pain. According to the the authors, TG neuronal silencing resulted in significantly reduced behavioral responses to vibrissal pad mechanical stimulation and decreased c-fos expression in the brainstem trigeminal nucleus caudalis [37].

### Other modulation approaches in TG

Apart from the approaches mentioned above, other modulation approaches include gamma knife radiosurgery (GKS), percutaneous radiofrequency (RF), and photobiomodulation (PBM) in TG. A study has postulated that given its short latency duration, low collateral risks, and a significant share of pain management, GKS on TG is indeed a feasible therapy choice. The inactivation of the satellite glial cells in the TG might well be associated with the action mechanisms of radiosurgery [42]. Radiosurgery in the TG induces a lesion to the SGC, which enables the degenerative mechanisms triggered by the affected neuron after a nerve injury to subside. An extracellular hyperpolarization that lasted longer could further result from reducing the overall SGCs' gaps and attributes. Clinically, pain discharges are inhibited and are sustained in this state for a considerable amount of time. Employing RF treatment of the TG for treating patients with TN has indicated high efficacy. However, there has also been a substantial level of adverse effects. Lower sensory stimulation was linked to more hypesthesia, whereas higher stimulation intensities were coupled with lower efficacy [43]. Another intriguing approach in TG was PBM, which centers on the dynamics of visible or near-infrared photons with the biomolecules present inside cells or tissues. In vitro studies revealed that effective PBM energy density, 1 and 2 J/cm2, and frequency, 10 Hz, promoted neuronal growth of TG neurons and might have therapeutic potential in circumstances prompting the regeneration of damaged TG neurons [44].

## Discussion

**Fig. 2:**
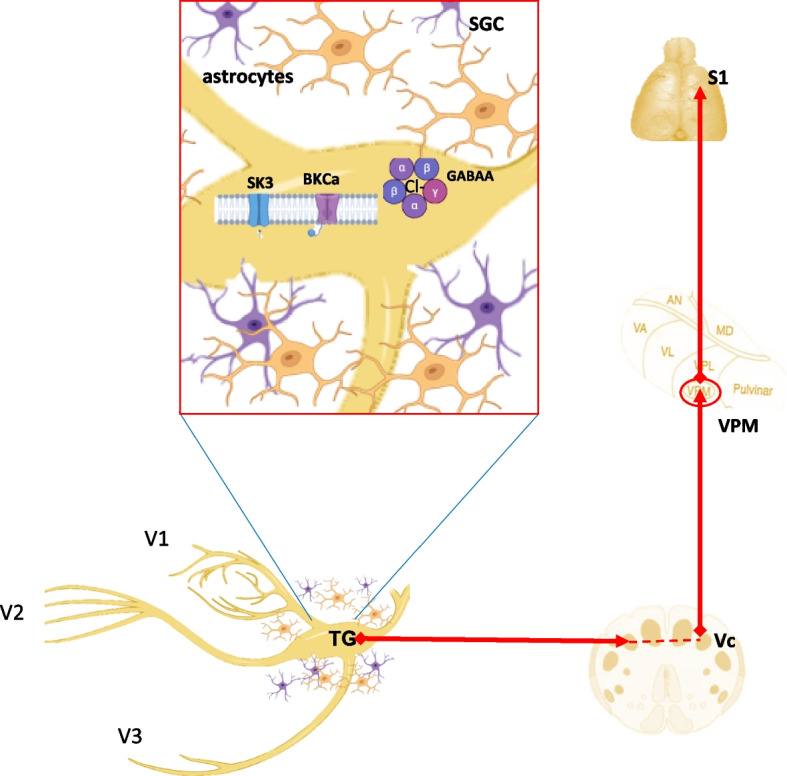
Synaptic connections of the trigeminal ganglion in the orofacial pain pathway. Three peripheral nerve branches (V1: ophthalmic; V2: Maxillary; V3; mandibular) of the trigeminal nerve, whose cell bodies sit in the trigeminal ganglion (TG), convey pain perception from the facial region and radiate centrally towards synapse with the second order neurons in the trigeminal spinal nucleus caudalis (Vc). Afterward, the second-order neurons ascend and end in the thalamus. Pain is mediated in the primary somatosensory cortex (S1), which receives nociceptive input from the thalamus. The maximized view of TG shows the prospects of targeting TG. Small-conductance calcium-activated potassium (SK) type 3 channel; Large conductance calcium-activated potassium (BKCa); γ-aminobutyric acid-A (GABAA) receptor; Satellite glial cells (SGC); VPM: Ventral posteromedial thalamus

The peripheral modulation of pain pathways in TN is facilitated by the trigeminal ganglion, a crucial element in the nociception of craniofacial pain. Trigeminal neuropathic pain syndromes, which are often less effectively treated with traditional surgical techniques like microvascular decompression and percutaneous rhizotomy, can be treated with TG stimulation, a neuromodulatory therapy. A study has reported that the trigeminal branch stimulation of the V3 dermatome carries a particularly significant risk of cutaneous erosion because of the lower jaw's extreme movement. Therefore, integrating TG stimulation, which also crosses the V3 distal nerve as it joins the ganglion, would be a feasible approach [32] (Fig. 2).

Another possibility is targeting multiple TG channel pathways.Essential sodium channel barriers that are believed to occur in the dorsal root ganglion because of nerve injury also occur in the TG [45]. TN may indeed be managed with newer drugs targeting sodium channel subtypes in TG. Small-conductance calcium-activated potassium (SK) type 3 channel is present in TG and might be involved in TN pathogenesis. Thus, targeting this channel in TG may lay the basis for TN therapy [46]. It has been reported that TG expresses many large conductance calcium-activated potassium (BKCa) channels, which are thought to contribute to the anti-hyperalgesic action in neuropathic pain. To better comprehend mechanical allodynia, a study that focused on TG neurons discovered that the downregulation of BKCa channels in both injured and intact nociceptive TG neurons was a key factor in the TN [47]. They proposed that manipulating brain natriuretic peptide or its receptor or BKCa channel pathway in TG corresponds to a novel therapeutic point for the clinical management of TN [48]. Because TG contains γ-aminobutyric acid (GABA) and its receptors, a study investigated the masticatory muscle-evoked brainstem trigeminal neuron responses following intraganglionic injection of muscimol (GABAA) but not baclofen (GABAB). It was found that activating GABAA receptors might have a gating function on sensory transmission via TG by boosting innocuous sensory input and lowering putative nociceptive input [49].

In contrast to targeting TG in humans, there are some difficulties in targeting TG in rodents for preclinical studies. Only a few techniques fordelivering drugs or viral vectors to rodents' trigeminal ganglia have been described. Among them are the cranial and infraorbital routes. By targeting TG from the base of the skull, the dura mater's impact on the viral vector's diffusion into the trigeminal ganglia is avoided. As a result, it shielded the brain and peripheral nerves, limiting any likelihood of neuropathic pain after the injection. Additionally, it has been proposed that thetransduction of CGRP shRNA may partially reduce discomfort following TG injections [50]. We followed the stereotaxic technique to inject the optogenetic virus into TG as mentioned in earlier studies [51, 52]. In addition, we employed flexible optic fiber to modulate the TG activity upon stimulation, which ensures high-quality coupling and durability [38]. Specific targeting and understanding of the molecular perspectives in trigeminal ganglion may help highlight the potential therapeutic intervention in orofacial neuropathic pain.

## Conclusion

Numerous brain areas have been investigated to date to modulate the trigeminal pain pathway. The trigeminal ganglion, a key component of the trigeminal pain circuitry, has also been studied using electrical, pharmacological, optogenetic, chemogenetic, radiological, and other methods. This review will help gain the new vision towards targeting TG directly for TN management.

## Data Availability

Not applicable.

## Abbreviations

BKCa: Large conductance calcium-activated potassium channels
CNO: Clozapine N-oxide
CGRP: Calcitonin gene-related peptide
Cdk5: Cyclin-dependent kinase 5
DREADD: Designer Receptors Exclusively Activated by Designer Drugs
DRG: Dorsal root ganglion
GABAA: γ-Aminobutyric acid A receptor
GFAP: Glial fibrillary acidic protein
GKS: Gamma knife radiosurgery
NpHR: Halorhodopsin from Natronomonas
OXTR: Oxytocin receptors
PACAP: Pituitary adenylate-cyclase-activating polypeptide receptor type 1
PBM: Photobiomodulation
SGC: Satellite glial cells
SK3: Small-conductance calcium-activated potassium type 3 channel
TG: Trigeminal ganglion
TN: Trigeminal neuralgia
TRP: Transient receptor potential
TRPV1: TRP vanilloid type 1 receptor channel
VIP: Vasoactive intestinal peptide
VPm: Ventral Posteromedial nucleus of the thalamus
